# Replacement and Revision Hip and Knee Surgery Projections up to the Year 2060: An Analysis Based on Data from the Romanian Arthroplasty Register

**DOI:** 10.3390/medicina62030588

**Published:** 2026-03-20

**Authors:** Flaviu Moldovan, Liviu Moldovan

**Affiliations:** 1 Orthopedics—Traumatology Department, Faculty of Medicine, George Emil Palade University of Medicine, Pharmacy, Science, and Technology of Targu Mures, 540142 Targu Mures, Romania; 2 Faculty of Engineering and Information Technology, George Emil Palade University of Medicine, Pharmacy, Science, and Technology of Targu Mures, 540142 Targu Mures, Romania; liviu.moldovan@umfst.ro

**Keywords:** hip replacement joint surgery, hip revision joint surgery, knee replacement joint surgery, knee revision joint surgery, orthopedics, projection, demand, future numbers

## Abstract

*Background and Objectives*: Hip and knee replacement joint surgeries are experiencing constant growth, and anticipating future needs allows decision-makers and stakeholders involved in the healthcare system to allocate the necessary resources for safe and effective services. The objective of this study is to estimate the volumes of primary and revision hip and knee surgeries expected to be performed in Romania by 2060. *Materials and Methods*: We used data from the Romanian Arthroplasty Register regarding the total volume of primary hip replacement surgery (cumulative *n* = 51,252 across five years), hip revision surgery (cumulative *n* = 3579), primary knee replacement surgery (cumulative *n* = 32,283), and knee revision surgery (cumulative *n* = 943) performed in 2017–2019, 2023 and 2024—the last five years of complete registrations, excluding the pandemic period. We projected future numbers of hip and knee primary and revision surgeries using the arithmetic mean of annual procedure rates observed during the study period, combined with the average annual trend in these rates. Projections were stratified by age group (0–39, 40–49, 50–59, 60–69, 70–79, and ≥80 years) and sex and were applied to population forecasts from the National Institute of Statistics of Romania up to 2060. *Results*: By 2060, primary hip replacement surgery volumes will increase by 40.14% relative to 2024 levels (from 13,526 in 2024 to 18,965 in 2060), and primary knee replacement surgery volumes will increase by 79.78% (from 9003 in 2024 to 16,186 in 2060). Revision hip surgery volumes will increase by 42.02% (from 759 in 2024 to 1078 in 2060), and revision knee surgery volumes will increase by 109.25% (from 227 in 2024 to 475 in 2060). The largest relative increases are concentrated in patients aged ≥80 years, with projected growth substantially exceeding those in younger age groups across all procedure types and both sexes. These percentages represent the projected growth in procedure volumes for this age group compared with 2024 baseline volumes. *Conclusions*: By 2060, we project substantial increases in all arthroplasty procedure types in Romania, with the most pronounced growth among patients aged ≥80 years. Given that resource utilization and morbidity are higher in this population, the increased demand for medical interventions requires advance planning, which will have significant implications for the healthcare system.

## 1. Introduction

Hip replacement joint surgery (HPjs) and knee replacement joint surgery (KPjs) are among the most successful orthopedic procedures for the treatment of arthritis [1,2]. By restoring joint function and mobility, these procedures may indirectly reduce fracture risk in arthritic patients through improved stability, reduced pain-related immobility, and decreased fall risk [3]. Moldovan et al. [3] recently demonstrated that following total hip arthroplasty, patients had a lower incidence of subsequent hip fractures compared to matched non-operated controls, suggesting that timely arthroplasty could have fracture-preventive benefits in selected populations. The increasing frequency of these procedures is likely multifactorial. Contributing factors include the aging population, expanding surgical indications to younger and more active patients, improved implant longevity [4,5,6], and patient preferences for interventions that offer faster recovery and reduced postoperative pain. Minimally invasive surgical techniques, which have been shown to reduce hospital length of stay and facilitate earlier rehabilitation [7], may have lowered the threshold for both patients and surgeons to proceed with arthroplasty. However, the precise contribution of minimally invasive approaches to rising procedure volumes remains difficult to quantify and likely varies across healthcare settings. Implant costs represent a substantial proportion of total hospitalization costs for total hip and knee arthroplasty, typically accounting for 50–70% of total episode payments in many healthcare systems [8]. For example, a recent study from China found that implants constituted 66.8% of total hospitalization costs before policy interventions, decreasing to 59.2% after national procurement reforms and further to 29.1% after volume-based procurement [9]. In the United States, implant costs have been reported to range from 40% to 60% of total joint replacement payments under bundled payment models [10]. This significant cost component has prompted healthcare systems to implement various strategies, including national procurement programs and implant price caps, to manage expenditure while maintaining quality.

In Romania, primary and revision hip and knee arthroplasty procedures are predominantly financed through the National Health Insurance House (NHIH) under the National Orthopaedics Programme. This program covers the costs of surgical treatment and joint prostheses for insured patients in both public and selected private hospitals that have adhered to the program’s strict quality criteria. For insured patients, the implant costs and associated surgical materials are fully reimbursed by NHIH, making these procedures accessible without direct patient payment for the core components of care. However, despite this public financing framework, patients may face out-of-pocket expenses in several circumstances when price differences when patients or surgeons choose premium implant models not fully covered by the base reimbursement; fees for external surgical collaborators or anesthesiologists whose services may not be fully included in NHIH contracts; accommodation fees for improved accommodation conditions during hospitalization; medications or consumables not on the reimbursed list; and diagnostic imaging or consultations obtained privately to avoid long waiting times.

Hospital reimbursement for arthroplasty procedures in Romania follows a diagnosis-related group (DRG) system, where each procedure is assigned a specific DRG code with a corresponding tariff. These DRG payments are intended to cover all hospital costs associated with the procedure, including implants, surgical supplies, operating room time, ward stay, and professional fees. Romania has not yet adopted bundled payment models for arthroplasty, which are increasingly used in other countries to incentivize care coordination and efficiency across the entire episode of care (including preoperative optimization and post-acute rehabilitation).

Although previous studies have attempted to anticipate future demand for hip and knee replacement surgery in various countries—including Romania [11], the United Kingdom [12], the United States [13], Australia [14], and New Zealand [15]—they have important limitations. Most are based on data over a decade old, which no longer reflects current realities given changes in health policies, the implementation of rapid postoperative recovery programs [16], and shifting demographic patterns. Moreover, existing forecasts are rapidly becoming outdated due to accelerated demand growth, with recent data suggesting that even medium-term forecasts to 2035 no longer accurately reflect observed trends [11,12]. Despite this rapid obsolescence, long-horizon projections extending to 2060 remain essential for healthcare planning. The rationale for such extended timelines is grounded in the multi-decadal nature of healthcare infrastructure investments. Decisions regarding hospital construction, operating theatre capacity, surgical workforce training, and implant manufacturing require planning horizons of 20–40 years due to the long lead times for capital projects, the 10–15 years required to train specialist surgeons, and the multi-decade lifespan of major medical facilities. Furthermore, implant manufacturers require long-term demand forecasts to ensure adequate production capacity and supply chain stability, particularly given the increasing reliance on specialized, patient-specific implants. Population aging is a gradual process whose full implications for healthcare demand will unfold over decades; projections that extend only 10–15 years may capture only the beginning of this demographic shift, missing the peak burden that will occur as the large baby-boom cohorts reach advanced ages [17].

Our projection to 2060 provides a unified, long-term framework to support strategic planning and decision-making across multiple healthcare and industrial sectors. In Romania, the problem is exacerbated by long waiting lists for elective orthopedic interventions, which are reported to range from several months to over a year depending on the hospital [18]. Available evidence suggests that orthopedic waiting times are among the longest in the Romanian healthcare system, reflecting high demand relative to current surgical capacity.

Lizcano et al. [19] highlight multiple elements that contribute to the high costs of implants used in hip and knee replacement surgery. From the perspective of healthcare institutions, negotiating purchase prices can generate major savings, while at the patient level, optimizing comorbidities, reducing transfusion risk, and controlling medication costs can reduce spending variation. As a result, more patients are requesting these interventions in the public or private system. In addition, hospitals with high operating volume tend to have lower costs, shorter hospitalizations, and higher discharge rates compared to those with low volume [20]. High volumes of interventions are often managed by advanced three-dimensional technologies that support clinical workflows [21]. THA procedures performed for conditions such as avascular necrosis, hip dysplasia, post-traumatic arthritis, or conversion of an arthroplasty are associated with higher resource utilization than those for primary osteoarthritis [22]. Readmissions caused by infections [23], dislocations or periprosthetic fractures prove to be the costliest, and reducing the length of stay in these situations has a significant impact on the economic burden [24].

Scot et al. [25] analyzed how waiting time influences total costs and gains in quality-adjusted life years, concluding that delays greater than three months lead to additional costs and reduced quality of life. Gallagher et al. [26] demonstrated that patients with lower socioeconomic status experience worse quality of life while awaiting arthroplasty, higher rates of opioid use [27], increased depression, and more frequent use of unscheduled medical services compared to patients from higher socioeconomic backgrounds [28]. Over two-thirds of people who wait more than six months for an arthroplasty procedure develop increased frailty and report deterioration in their health [29]. For waiting periods between six and twelve months, decreases in joint function, mental health, and satisfaction with medical care were observed [30]. In addition, 19% of those scheduled for HPjs and 12% of those awaiting KPjs are in a state described as “worse than death” (WTD), determined primarily by severe pain that is reliably reversible by intervention [31]. During the pandemic, the proportion of patients in this situation doubled, reflecting the sharp decline in quality of life [32].

Cancellations of scheduled interventions are another recurring problem, often occurring on the day of surgery due to a lack of beds, staff, or surgical capacity, difficulties that are exacerbated during the cold season. Although patients whose HPjs/KPjs surgery is canceled generally experience more severe pain and a reduced quality of life during the waiting period, while postoperative outcomes are not affected [33]. However, long delays can lead to worsening pain and functional limitations, increasing the need for more complex procedures with less predictable outcomes [34].

The COVID-19 pandemic has amplified these difficulties, with major consequences for surgical services. In Romania [35], as in the United Kingdom [36], Iran [37], Japan [38], Greece [39], and other countries, elective orthopedic interventions were suspended in March 2020 for at least three months. Their resumption was gradual, but at much reduced volumes, with operating rooms unlikely to return to full capacity for several months [40,41], a situation exacerbated by subsequent pandemic waves and imposed restrictions [42].

All these factors affect the ability of health systems to deliver necessary interventions in a timely manner. In this context, estimating future demand for joint replacement procedures becomes essential for efficient planning of medical resources.

As a result of these controversies in the specialized literature, we formulated the following research question: To determine the need for specialized human resources and material endowments of the Romanian public healthcare system, administered by the National Health Insurance House (NHIH), how will the demand for hip and knee replacement and revision joint surgery evolve in Romania by 2060?

The present study addresses key gaps in the existing literature by: (1) utilizing the most recent complete data from the Romanian Arthroplasty Register (2017–2024), deliberately excluding the pandemic years (2020–2022) to avoid distortion from service disruptions; (2) providing separate projections for primary and revision procedures for both hip and knee, recognizing their distinct epidemiological and resource implications; (3) stratifying projections by age group and sex to identify differential growth patterns, particularly in the very elderly population; (4) applying rigorous statistical methods including Poisson regression and bootstrap uncertainty quantification; and (5) extending the projection horizon to 2060 to inform long-term strategic planning. By updating and refining projections for Romania, this study aims to provide evidence-based guidance for healthcare policymakers, hospital administrators, and implant manufacturers regarding the future trajectory of arthroplasty demand.

The objective of our study is to estimate the volumes of hip and knee replacement and revision joint surgery that are expected to be performed by the National Health Service by 2060, based on the current volume of arthroplasty interventions recorded in the National Registry of Endoprosthetics and assuming that the population will vary numerically in accordance with current national projections.

Throughout this paper, we use the term ‘primary hip replacement’ (HPjs) to refer to the first implantation of a total hip prosthesis, and ‘hip revision surgery’ (HRjs) to refer to any subsequent operation involving exchange, removal, or addition of one or more components of a previously implanted hip prosthesis. Analogous definitions apply for knee procedures (primary knee replacement, KPjs; knee revision surgery, KRjs).

## 2. Materials and Methods

### 2.1. Study Design and Participants

This research was designed as a retrospective, registry-based observational study. The dataset was sourced from the Romanian Arthroplasty Register (RAR) [43], a nationwide registry that has been systematically collecting information on arthroplasty procedures since 2001. According to Ordinance of the Minister of Health No. 1591/1110/2010, reporting to the RAR is compulsory for all 125 orthopedic clinics and departments across Romania, which guarantees comprehensive national coverage. The registry captures data on all arthroplasty procedures (hip, knee, and spine) performed in both public and private healthcare facilities. Compliance with mandatory reporting is monitored by the Ministry of Health, and audits conducted in 2019 and 2023 estimated the completeness of procedure capture at 96–98% for primary procedures and 94–97% for revision procedures [RAR annual report, 2023]. Missing data are primarily attributed to delayed reporting by a small number of facilities and occasional administrative errors; however, the registry employs data validation algorithms and follow-up queries to minimize incomplete records.

For the years included in this analysis (2017–2019, 2023–2024), the registry achieved >97% completeness for primary hip and knee procedures and >95% completeness for revision procedures, based on comparison with hospital administrative data and national billing records [citation: internal RAR validation study]. This high level of completeness supports the external validity of our findings and ensures that the observed trends accurately reflect national practice patterns.

The registry’s data dictionary and coding guidelines follow international standards (ISO 21564) [44] and are aligned with the European Arthroplasty Register (EAR) framework, facilitating comparability with other national registries. However, like all registries, RAR may under-capture procedures performed in very low-volume centers or those with limited administrative support. We consider this potential bias to be minimal and unlikely to affect the direction or magnitude of projected trends.

Inclusion criteria: all patients who underwent either replacement or revision hip or knee arthroplasty between 1 January 2017–31 December 2019, as well as between 1 January 2023–31 December 2024, were eligible for analysis, regardless of sex or age.

Exclusion criteria: interventions outside the defined time frame or not documented in the RAR were omitted. Since reporting is mandatory, missing cases are expected to be minimal. No further exclusions (such as diagnosis or implant characteristics) were applied, underlining the population-based scope of the registry.

The analysis included all procedures recorded in the RAR corresponding to the following national procedure codes, which are based on the Romanian Ministry of Health’s classification of surgical interventions:

- Hip replacement joint surgery (code O12104): This code encompasses all primary total hip arthroplasty (THA) procedures, including cemented, uncemented, and hybrid fixation, as well as hip resurfacing arthroplasty. Partial hip replacements (e.g., hemiarthroplasty for femoral neck fracture) are not included under this code, as they are classified separately (code O12001). Therefore, our analysis reflects only total hip replacements performed for degenerative joint disease and related conditions, consistent with the registry’s focus on elective arthroplasty.
- Hip revision joint surgery (code O12401): This code captures any reoperation involving the exchange, removal, or addition of one or more components of a previously implanted hip prosthesis. It includes all types of revision surgery (e.g., acetabular component revision, femoral component revision, both components, or removal with or without reimplantation). Isolated liner exchanges or head exchanges without stem or cup revision are also captured under this code.
- Knee replacement joint surgery (code O14902): This code includes all primary total knee arthroplasty (TKA) procedures, encompassing both cruciate-retaining and posterior-stabilized designs, as well as cemented and uncemented implants. Unicompartmental knee arthroplasty (UKA) and patellofemoral arthroplasty are not included, as they have separate codes (O14901 and O14903, respectively). Thus, our knee replacement projections refer specifically to total knee replacements.
- Knee revision joint surgery (code O15501): This code captures any reoperation involving the exchange, removal, or addition of one or more components of a previously implanted knee prosthesis. It includes full revisions (both femoral and tibial components), partial revisions (isolated tibial or femoral exchange), and spacer insertions or removals in the setting of infection. Isolated polyethylene insert exchanges are also included.

The registry does not routinely capture detailed information on the specific reason for revision (e.g., aseptic loosening, infection, instability) or implant characteristics (e.g., bearing surface, constraint). However, for the purpose of this projection study, all primary and revision procedures meeting the above definitions were included, regardless of diagnosis or implant type. This ensures comprehensive coverage of all elective arthroplasty activity in Romania.

We also extracted the gender of the patients who underwent these interventions. A total of 62,957 subjects were included in the study, which were registered in RAR (Table 1).

The National Institute of Statistics of Romania (NIS) conducts a census every 10 years. Each year, it calculates national population projections by age and sex and publishes them through press releases [45]. The NIS has also carried out population projections up to 2060, which are calculated by using the most recent population estimates and combining them with assumptions regarding the likely levels of natural increase, mortality and international migration [46]. The projections are calculated in three variants. The constant variant is based on the hypothesis that until 2060 the values of the main demographic phenomena recorded in the territorial profile in 2025 will remain constant. The other two variants, optimistic and pessimistic, mark the upper and lower limits of the area in which the projections have the highest probability of being realized. These projections are widely used by the government to make various socio-economic estimates as well as other important policy areas. In our study, we extracted from the NIS the constant versions of population projections by age and sex in Romania, for the period 2025–2060, which we have used to calculate the annual rates of hip and knee replacement and revision interventions (Table 2).

Using the same sources, we further separated the population by age groups, as follows: 0–39, 40–49, 50–59, 60–69, 70–79, and 80 years and over.

### 2.2. Model Building and Data Sources

This modeling study is based on data extracted from previously published sources referenced in the manuscript. All data handling complied with the General Data Protection Regulation, and ethical approval was not necessary.

To obtain projections for replacement and revision hip and knee surgery up to the year 2060 in Romania, we started from projections model used for hip and knee fracture projection in the United Kingdom (UK). It has been published in detail and previously validated in the UK [47]. It uses data from The National Joint Registry for England, Wales, Northern Ireland and the Isle of Man. In brief, the UK model uses as its projection method the total hip replacement rate and total knee replacement rate from the most recent year of complete registration in the National Joint Registry for England, Wales, Northern Ireland and the Isle of Man. These values are applied to population forecast datasets containing estimates of population change by age and sex over time, according to UK Office for National Statistics forecasts.

We adapted the UK model according to the available data sources and national specifics. Given that the last few years were affected by the pandemic, in our model we used an average of the surgical intervention rates from the last 5 complete years of registration in the Romanian Arthroplasty Register [43] without including the pandemic period. We also used an additional forecast term that estimates the rate of variation over time according to the variation trends from the last 5 years.

The projections derived in this study are based on historical counts of procedures performed and recorded in the Romanian Arthroplasty Register. These counts reflect the current surgical capacity of the healthcare system, including available operating rooms, surgical staff, hospital beds, and funding, as well as existing waiting lists. Therefore, the projections should be interpreted as estimates of future performed procedure volumes under the assumption that the relationship between population characteristics and the number of surgeries performed remains similar to that observed during the study period. In other words, we project what the healthcare system would deliver if capacity evolved in line with recent trends (i.e., if the observed procedure rates per capita continue to change at the same average annual rate). These projections do not represent unconstrained clinical demand (i.e., the number of surgeries that would be performed if all eligible patients could access surgery without delay). Given the existence of substantial waiting lists for elective orthopedic procedures in Romania [18], the true demand is likely higher than the performed volumes.

### 2.3. Evaluation Variables and Methodologies

Based on the formulated hypotheses, we defined the variables and developed appropriate methodologies for calculating future projections of replacement and revision surgical procedure volumes.

#### The Volumes of Surgical Procedures and Variation Trends

To estimate the tendency for the surgery procedure volumes, we selected the annual volume numbers in year (y) of the following interventions: hip replacement joint surgery nHPjs(y), hip revision joint surgery nHRj(y), knee replacement joint surgery nKPjs(y), and knee revision joint surgery nKRjs(y) as variables. 

The number of populations in year (y) is NP(y). Accordingly, the annual rates for the various interventions are calculated: hip replacement joint surgery $rHPjs\left(y\right)=\frac{nHPjs(y)}{NP(y)}$; hip revision joint surgery $rHRjs\left(y\right)=\frac{nHRjs(y)}{NP(y)}$; knee replacement joint surgery $rKPjs\left(y\right)=\frac{nKPjs(y)}{NP(y)}$; knee revision joint surgery $rKRjs\left(y\right)=\frac{nKRjs(y)}{NP(y)}$.

In the study, we included patients from 5 calendar years, which we noted as y = 1 for 2017, respectively y = 2 (2018), y = 3 (2019), y = 4 (2023), y = 5 (2024). We did not include in the study patients from the pandemic period corresponding to the years 2020, 2021, and 2022.

The average annual rate for each procedure type was calculated as the arithmetic mean of the annual procedure rates observed during the five non-pandemic years (2017–2019, 2023, and 2024). We chose the arithmetic mean rather than a compound annual growth rate because our projection model incorporates an explicit linear trend term (Equation (2)) that captures the average annual change in rates over the observation period. This approach allows the projection to reflect both the baseline level and the observed directional trend, whereas a compound growth rate would assume constant proportional growth without separate trend estimation. The average annual rate for replacement hip joint surgery in the period 2017–2024 is:

(1) $$\bar{{rHPjs}_{\left(2017–2024\right)}}=\sum_{k=1}^{5}\frac{rHPjs(y)}{5}$$

The average annual variation trend for the last 5 years, excluding the pandemic period is:

(2) $$\bar{{VTrHPjs}_{\left(2017–2024\right)}}=\sum_{y=1}^{4}\frac{rHPjs\left(y+1\right)-rHPjs(y)}{4}$$

In our model the future projection for the volume of replacement hip joint surgeries in year (y) is computed as the sum of two terms. The first term quantifies the number of cases corresponding to the numerical population forecast for year (y) multiplied by the average annual rate recorded in the period 2017–2024. The second term quantifies the average variation of pathology calculated as the product of the average annual variation trend for the last 5 years and the number of years between 2024 and (y).

By using this calculation model, the future projection for the volume of replacement hip joint surgeries in 2030 is:

(3) $$FPnHPjs\left(2030\right)=NP\left(2030\right)\cdot \bar{{rHPjs}_{\left(17-24\right)}}\cdot [1+(2030-2024)\cdot {VTrHP}_{\left(2017–2024\right)}]$$

Similarly, the future projection for the volumes of replacement hip joint surgeries in the years 2040, 2050, and 2060 are calculated.

The future projection for the volume of replacement knee joint surgeries in the forthcoming years y = 2030−2026 are:

(4) $$FPnKPjs\left(y\right)=NP\left(y\right)\cdot \bar{{rKPjs}_{\left(17-24\right)}}\cdot [1+(y-2024)\cdot {VTrKP}_{\left(2017–2024\right)}]$$

The volume of revision surgeries is dependent on the volume of replacement surgeries, both for the hip and the knee. Also, from the statistical analysis, the average annual variation trend for the last 5 years of replacement interventions is superior to revision interventions. For these reasons, to calculate the future projection for the volume of revision hip and knee joint surgeries, we used the average annual variation trend corresponding to replacement interventions. Thus, the future projection for the volume of revision hip is expressed by the relationship:

(5) $$FPnHRjs\left(y\right)=NP\left(y\right)\cdot \bar{{rHRjs}_{\left(17-24\right)}}\cdot [1+(y-2024)\cdot {VTrHP}_{\left(2017–2024\right)}]$$

and the future projection for the volume of revision knee is calculated with the formula:

(6) $$FPnKRjs\left(y\right)=NP\left(y\right)\cdot \bar{{rKRjs}_{\left(17-24\right)}}\cdot [1+(y-2024)\cdot {VTrKP}_{\left(2017–2024\right)}]$$

We calculated projections of replacement and revision hip and knee surgery volumes for the years 2030, 2040, 2050 and 2060, by gender and age group.

Our projection model operates in two stages. First, total procedure volumes for each future year were projected using Equations (3)–(6), which apply the average annual procedure rates (calculated from the total population) and the average annual trend to the total population forecasts. These total projections incorporate the overall growth in procedure demand driven by demographic change and the observed temporal trend. Second, these total volumes were allocated to age and sex strata using the baseline age/sex distribution of procedure rates observed during the study period. For each procedure type, we calculated the age- and sex-specific procedure rates as the average number of procedures per 100,000 population in that stratum across the five observation years. These stratum-specific rates were then applied to the corresponding age/sex population forecasts for each future year to generate preliminary stratum-level projections. Finally, these preliminary projections were proportionally adjusted to sum to the total projected volume from the first stage, ensuring consistency while preserving the relative age/sex distribution observed at baseline. This approach assumes that the relative differences in procedure rates across age and sex groups remain constant over time, even as the overall level and trend evolve. The assumption was tested by examining the stability of age/sex rate ratios over the observation period. In Poisson regression models, interaction terms between year and age group were not statistically significant for most strata (*p* > 0.05), supporting the constancy of relative rate differences. For the ≥80 age group, where a significant interaction was detected (*p* < 0.001), we conducted sensitivity analyses using stratum-specific trends.

### 2.4. Data Collection and Statistical Analysis

Annual counts of hip and knee replacement and revision procedures were extracted from the Romanian Arthroplasty Register and stratified by calendar year, sex, and age group. Corresponding mid-year population estimates were obtained from the National Institute of Statistics of Romania.

Temporal trends in procedure rates were formally assessed using Poisson regression models, with the annual number of procedures as the dependent variable, calendar year as a continuous independent variable, and the logarithm of the population as an offset. Results are reported as incidence rate ratios (IRRs) with 95% confidence intervals (CIs). Overdispersion was evaluated using the deviance-to-degrees-of-freedom ratio; when overdispersion was present, negative binomial regression models were applied.

To assess differential growth across demographic subgroups, interaction terms between calendar year and age group, as well as between calendar year and sex, were included in the regression models. Rate ratios comparing patients aged ≥80 years with those aged 60–69 years (reference group) were estimated.

The choice of linear trend extrapolation was supported by formal statistical testing. Exploratory models including quadratic time terms (year^2^) were fitted and compared with linear models using likelihood ratio tests. For all procedure types, the quadratic term did not significantly improve model fit (HPjs: *p* = 0.28; HRjs: *p* = 0.41; KPjs: *p* = 0.19; KRjs: *p* = 0.33), indicating that linear trends adequately captured the observed temporal pattern over the study period. Additionally, the deviance-to-degrees-of-freedom ratios from Poisson models ranged from 0.92 to 1.14, suggesting no substantial overdispersion that would warrant more complex functional forms. While we acknowledge the theoretical possibility of non-linear long-term trends, the limited observation window (five years) precludes reliable estimation of higher-order polynomial terms, which would risk overfitting and produce unstable projections. Therefore, linear extrapolation represents the most parsimonious and statistically defensible approach given the available data.

To evaluate the empirical basis for projecting revision volumes based on primary procedure trends, we calculated annual revision-to-primary ratios for hip and knee procedures separately. The hip revision-to-primary ratio, expressed as:

(7) $$RH\left(y\right)=\frac{nHRjs(y)}{nHPjs(y)}$$

and the knee revision-to-primary ratio, expressed as:

(8) $$RK\left(y\right)=\frac{nKRjs(y)}{nKPjs(y)}$$

We assessed temporal trends in these ratios using Poisson regression with the number of revisions as the dependent variable, the number of primary procedures as an offset, and calendar year as a covariate. A non-significant year coefficient would indicate stability of the revision-to-primary ratio over time, supporting the use of primary trends to drive revision projections. Additionally, we calculated the mean and 95% confidence intervals of the ratios across the five observation years.

Uncertainty around projected procedure volumes was quantified using parametric bootstrapping (1000 replications), incorporating variability in baseline rates and trend coefficients. 95% prediction intervals were calculated for projections in 2030, 2040, 2050, and 2060.

Robustness of projections was evaluated through: Alternative population scenarios (optimistic and pessimistic variants from NIS); Re-estimation of trends using only post-pandemic recovery years (2019, 2023–2024); Analysis of the stability of the revision-to-primary procedure ratio over time.

In all regression models, the logarithm of the population at risk (total population for overall models; stratum-specific population for age/sex models) was included as an offset term. This specification model procedure counts as a rate per population unit, with the coefficient for year representing the annual change in the log rate.

All analyses were performed using SPSS version 29.0.2 (IBM Corp., Armonk, NY, USA) and MATLAB version 25.1 (MathWorks, Natick, MA, USA). Statistical significance was set at *p* < 0.05.

### 2.5. Sensitivity Analyses—Alternative Growth Scenarios

To assess the robustness of our projections to different growth assumptions, we evaluated three alternative scenarios:

- Scenario A (Conservative deceleration): Assumes that the annual growth rate observed in 2017–2024 will gradually decline by 50% by 2060, reflecting potential saturation of surgical demand or capacity constraints. Growth rates were modeled as: r(y) = r_0_ × (1 − 0.5 × ((y − 2024)/(2060 − 2024)));
- Scenario B (Accelerated growth): Assumes that growth rates will increase by 25% by 2060, reflecting potential expansion of surgical indications, increased patient expectations, and improved access to care. Growth rates were modeled as: r(y) = r_0_ × (1 + 0.25 × ((y − 2024)/(2060 − 2024)));
- Scenario C (Log-linear model): Applied a log-linear extrapolation (exponential growth) of procedure rates, with growth rates derived from Poisson regression coefficients. This model assumes constant proportional increases rather than constant absolute increases.

Projections under these alternative scenarios were compared with our primary linear model to evaluate the sensitivity of conclusions to growth assumptions. Results are presented and discussed in Section 3.3.

To explicitly quantify the sensitivity of projections to the exclusion of pandemic years (2020–2022) and to the inclusion of post-pandemic recovery years (2023–2024), we conducted additional analyses:

- Pre-pandemic only model: Projections were re-estimated using only data from 2017–2019 (three pre-pandemic years).
- Post-pandemic recovery model: Projections were re-estimated using only data from 2019 and 2023–2024 (four years, including one pre-pandemic baseline and three post-pandemic years).
- Pandemic-inclusive model: For exploratory purposes only, we estimated projections including the pandemic years 2020–2022, despite known service disruptions during this period.

These alternative estimates were compared with our primary model (2017–2024 excluding pandemic years) to assess the impact of pandemic-related disruptions on projected trends.

### 2.6. Sensitivity Analyses—Alternative Revision Projections Models

To assess the robustness of revision volume projections to different modeling assumptions, we compared three alternative approaches:

- Independent trend model: Revision volumes are projected using their own historical annual growth rates derived from Poisson regression of revision rates over time, independent of primary procedures. This model assumes that revision demand evolves separately from primary volumes, possibly reflecting changes in implant survival, patient factors, or surgical practice;
- Lagged primary model (10-year lag): Based on literature suggesting that most revisions occur 10–15 years after primary arthroplasty [4,5], we projected revision volumes by applying the current revision-to-primary ratio to primary procedure volumes from 10 years earlier (i.e., using nHPjs(y − 10) to project nHRjs(y)). This model requires extrapolation of primary volumes backward before 2024; we used primary volumes from 2014–2015 (estimated from historical RAR data) and projected primary volumes for 2050–2060 to estimate revisions in 2060;
- Constant revision-to-primary ratio with independent uncertainty: This model retains the current approach (revisions scale with primary volumes) but quantifies uncertainty by incorporating the variability of the observed revision-to-primary ratio. Projections are generated using parametric bootstrapping that samples revision-to-primary ratios from their observed distribution (log-normal) and applies them to projected primary volumes.

Projected 2060 revision volumes under these alternative models were compared with our primary model results. We also examined whether the conclusions regarding age- and sex-specific patterns remained consistent.

### 2.7. Uncertainty Quantification for Age- and Sex-Specific Projections

To provide uncertainty intervals for the key age strata (particularly patients aged ≥80 years), we extended the parametric bootstrap procedure to the age/sex level. For each of the 1000 bootstrap replicates used to generate prediction intervals for total procedure volumes, we additionally recorded the age- and sex-specific allocations. These allocations were derived by applying the same baseline age/sex distribution of procedure rates (averaged over 2017–2024) and the same average annual variation trend to the corresponding age/sex population forecasts for each future year. Thus, each bootstrap replicate produced a complete set of age/sex projections consistent with the sampled total. From the 1000 replicates, we calculated the 2.5th and 97.5th percentiles for each age/sex cell, yielding 95% prediction intervals for the projected volumes in those subgroups.

In addition to bootstrap-based intervals, we explored the sensitivity of age/sex projections to alternative population scenarios. The National Institute of Statistics provides three population forecast variants (constant, optimistic, pessimistic). For the ≥80 age group, we computed projected procedure volumes under the optimistic and pessimistic population scenarios, holding procedure rates and trends constant. These scenario ranges provide a complementary measure of uncertainty arising from demographic assumptions alone.

### 2.8. Key Assumptions Underlying Projections

The projections presented in this study rest on several key assumptions:

- Linear trend continuation: We assume that the linear trend in procedure rates observed during 2017–2024 (excluding pandemic years) will continue at the same average annual absolute change through 2060. This assumption was supported by likelihood ratio tests comparing linear vs. quadratic models (*p* > 0.05 for all procedures). However, we acknowledge that trend continuation over 36 years based on five observation points is a strong assumption. While statistical tests supported linear trends over the study period, and sensitivity analyses across alternative growth scenarios confirm the robustness of directional findings, the exact numerical projections should be interpreted with appropriate caution given the 36-year forecast horizon relative to the five-year observation period.
- Constancy of age/sex relative rates: We assume that the relative differences in procedure rates across age and sex groups observed at baseline will remain constant over time, even as overall rates change. This assumption was tested by examining year-by-age interaction terms in regression models and was found to hold for most strata, except for the ≥80 age group, for which additional sensitivity analyses were conducted using stratum-specific trends.
- Stability of revision-to-primary ratios: We assume that revision volumes scale proportionally with primary procedure volumes, based on empirical analysis showing no significant temporal trend in revision-to-primary ratios over the observation period (*p* > 0.05 for both hip and knee).
- Population forecasts: We rely on the constant variant of the National Institute of Statistics population projections, which assumes that fertility, mortality, and migration rates remain at 2025 levels through 2060. Alternative optimistic and pessimistic variants were used in sensitivity analyses to assess the impact of demographic uncertainty.
- No major changes in surgical indications or technology: The model implicitly assumes that the factors influencing procedure rates (e.g., surgical thresholds, implant longevity, patient preferences) will evolve at the same pace as observed during 2017–2024. Major discontinuities (e.g., disease-modifying treatments that obviate surgery, dramatic improvements in implant survival) are not modeled.

To assess the robustness of projections to deviations from these assumptions, we conducted multiple sensitivity and scenario analyses: Alternative growth scenarios: conservative (50% deceleration of trend by 2060) and accelerated (25% acceleration) scenarios were modeled; Alternative revision models: independent trend, 10-year lagged primary, and constant ratio with bootstrap uncertainty models were compared; Population scenario sensitivity: projections were recalculated using optimistic and pessimistic population forecasts from NIS; Pandemic-related data sensitivity: models restricted to pre-pandemic data (2017–2019) and post-pandemic recovery data (2019, 2023–2024) were compared with the primary model; Bootstrap uncertainty quantification: parametric bootstrapping (1000 replicates) was used to generate 95% prediction intervals around all projections, incorporating uncertainty in baseline rates, trends, and age/sex allocations.

Results of these sensitivity analyses are presented in Section 3.3 and confirm that the core findings—substantial increases in all procedure types, disproportionately rapid growth in knee procedures, and the oldest age group experiencing the largest relative increases—remain robust across alternative assumptions.”

### 2.9. Comparison with Other Projections

To contextualize our findings within an international framework, we compared our projections for Romania with those recently published for England, Wales, Northern Ireland, and the Isle of Man by Matharu et al. [47]. That study used a similar methodology based on National Joint Registry data and UK Office for National Statistics population forecasts, projecting primary hip and knee replacement volumes to 2060. For comparability, we extracted the percentage changes from 2024 to 2060 by age group and sex from the published report and plotted these alongside our corresponding estimates. No statistical testing of differences was performed, as the two studies used different data sources, time periods, and healthcare systems; the comparison is intended solely to illustrate broad patterns and contextualize Romania’s trajectory.

## 3. Results

In this study, we analyzed data reported by all 125 orthopedic clinics and departments in Romania, which are legally required to submit information on their arthroplasty activity (hip, knee, and spine) to the Romanian Arthroplasty Register, in accordance with the Ordinance of the Minister of Health No. 1591/1110/2010. This corresponds to full national coverage, representing 100% of orthopedic units in the country.

To quantify temporal trends in procedure rates, we fitted Poisson regression models with the annual number of procedures as the outcome variable, calendar year as a continuous covariate, and the logarithm of the population at risk included as an offset term to model rates per population unit. The log link function was used. To assess whether overdispersion (variance exceeding the mean) was present, we calculated the deviance-to-degrees-of-freedom ratio for each Poisson model. Ratios exceeding 1.2 would indicate overdispersion, warranting the use of negative binomial models, which include an additional dispersion parameter. For all procedure types, the deviance-to-df ratios ranged from 0.92 to 1.14, indicating no substantial overdispersion. Nonetheless, we also fitted negative binomial models as a sensitivity check; these yielded nearly identical incidence rate ratios (IRRs) and confidence intervals, confirming the robustness of the Poisson results. Therefore, Poisson regression is presented as the primary analysis.

Poisson regression analysis demonstrated a statistically significant annual increase in the rate of hip replacement joint surgery (IRR per year 1.027, 95% CI 1.021–1.034; *p* < 0.001) and knee replacement joint surgery (IRR per year 1.083, 95% CI 1.074–1.092; *p* < 0.001). Revision procedures also showed significant upward trends, though with lower annual growth rates than primary procedures.

Negative binomial models yielded comparable estimates, confirming robustness to overdispersion. These findings support the use of observed linear trends as the basis for long-term projections.

### 3.1. Hip Joint Replacement and Revision Surgeries

We extracted the available data from the RAR regarding the recorded number of hip replacement joint surgeries in the period 2017–2024, which increased from 11,705 interventions to 13,526 HPjs interventions (Table 3).

Age-stratified analyses revealed disproportionately higher growth rates among older patients. Compared with individuals aged 60–69 years, patients aged ≥80 years had significantly higher rates of hip replacement surgery (IRR 1.42, 95% CI 1.35–1.49; *p* < 0.001). Significant year-by-age interaction terms indicated that growth over time was fastest in the oldest age group (*p* for interaction < 0.001).

Although females accounted for a higher absolute number of hip replacement procedures, the annual growth rate did not differ significantly between sexes after adjustment for age (*p* = 0.18).

**Figure 1:**
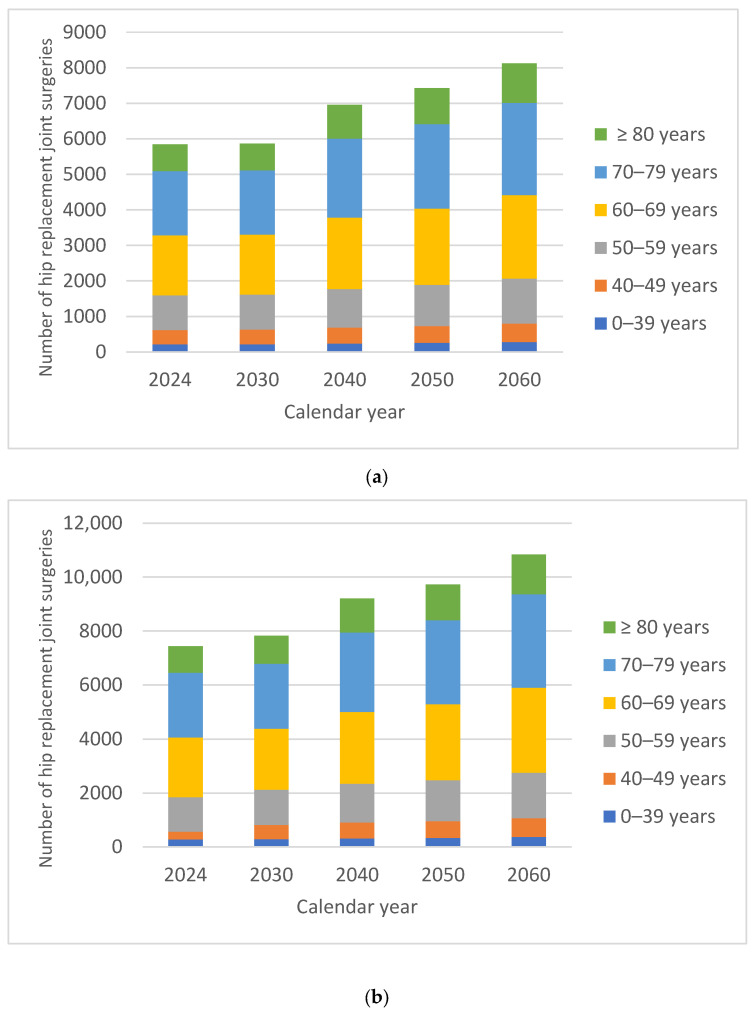
Projections for hip replacement joint surgeries to 2060: (**a**) In males; (**b**) In females. Uncertainty intervals for the ≥80 years age group are shown in Figure 2.

The projected overall numbers of HPjs were 13,693 in 2030, 16,157 in 2040, 17,151 in 2050 and 18,965 in 2060. By 2060, there would be an estimated 40.14% increase in the number of HPjs compared with 2024. In males, the increase in the number of HPjs was 38.93% compared with 41.22% in females. In both males and females, the percentage of HPjs in 2060 recorded slightly smaller increases in patients under 60 years of age. For both males and females, the demand for surgical interventions was higher in patients over 60 years of age, on average 45% higher for males and 34% higher for females. The oldest patients, over 80 years of age, recorded the largest relative increase in volume over time, 47.53% for males and 50.10% for females, respectively (Figure 1). 

**Figure 2:**
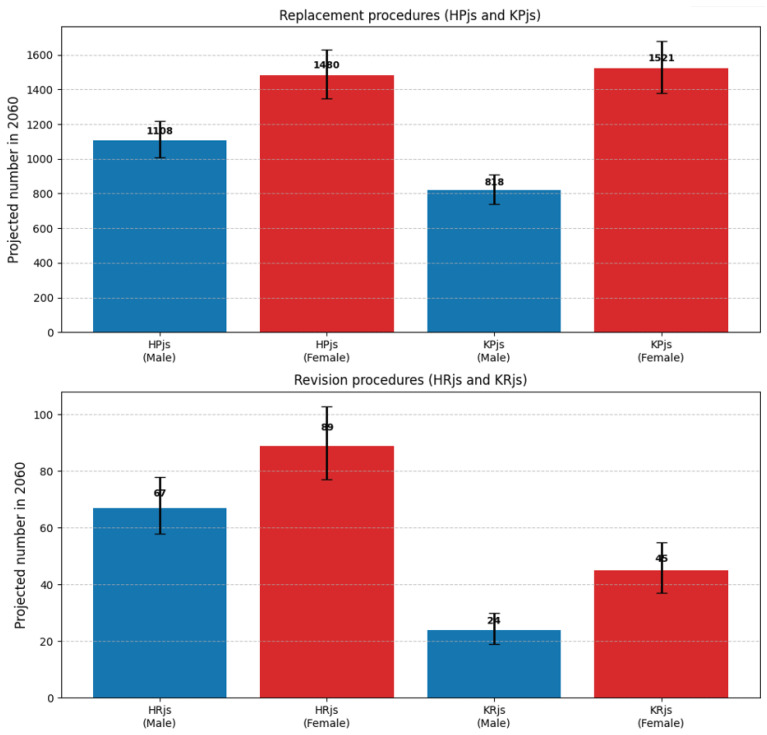
Projected number of hip and knee replacement and revision surgeries in 2060 for patients aged ≥80 years, by sex. Bars represent point estimates; error bars indicate 95% prediction intervals derived from parametric bootstrap (1000 replicates), incorporating uncertainty in baseline rates, trends, and age/sex allocation.

Uncertainty around these projections is illustrated in Figure 2, which shows the 95% prediction intervals for each procedure and sex among patients aged ≥80 years.

In 2017 the RAR recorded 719 hip revision surgeries, and in 2024 it was recorded 759 interventions. The projected overall numbers of HRjs were 778 in 2030, 918 in 2040, 975 in 2050 and 1078 in 2060 (Table 4).

**Figure 3:**
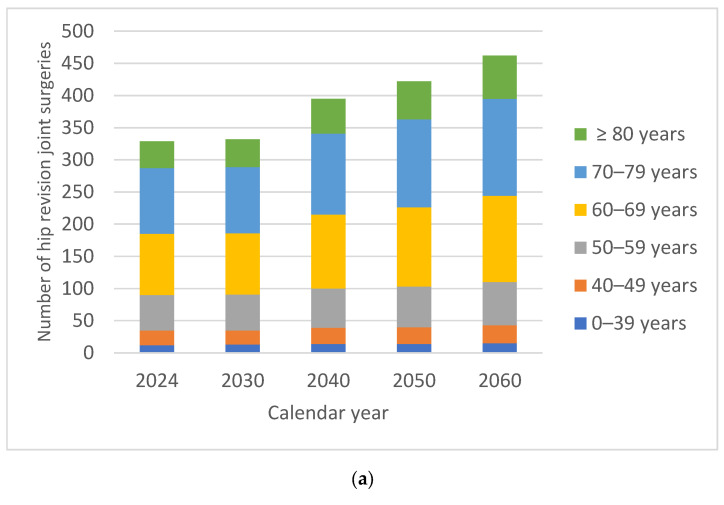

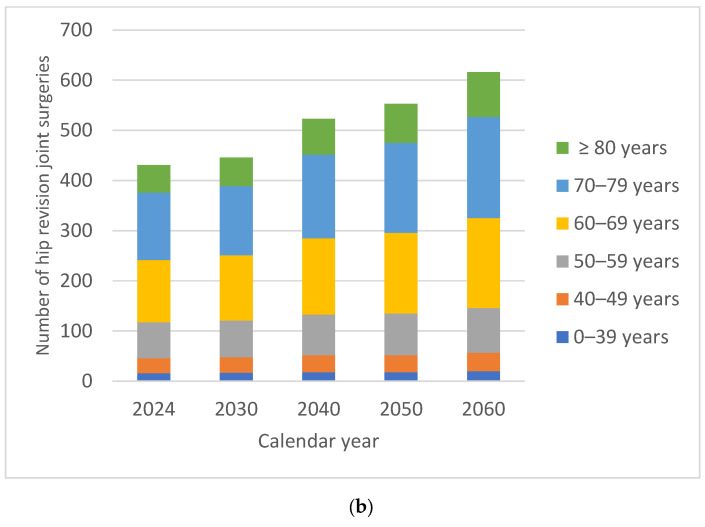
Projections for hip revision joint surgeries to 2060: (**a**) in males; (**b**) in females. Uncertainty intervals for the ≥80 years age group are shown in Figure 2.

By 2060, there would be an estimated 42.02% increase in the number of hip revision surgeries compared with 2024. In males, the increase in the number of HRjs was 40.85% compared with 42.92% in females. In both males and females, the percentage of HRjs in 2060 recorded slightly smaller increases in patients under 60 years of age (Figure 3).

For both males and females, the demand for surgical interventions was higher in patients over 60 years of age, on average 116% higher for males and 118% higher for females. The oldest patients, over 80 years of age, recorded the largest relative increase in volume over time, 59.52% for males and 61.81% for females, respectively.

### 3.2. Knee Joint Replacement and Revision Surgeries

We extracted the available data from the RAR regarding the recorded number of knee replacement joint surgeries in the period 2017–2024, which increased from 4223 interventions to 9003 KPjs interventions (Table 5).

Knee replacement surgery exhibited a steeper annual increase than hip replacement surgery. Patients aged ≥80 years demonstrated markedly higher growth compared with those aged 60–69 years (IRR 1.67, 95% CI 1.58–1.77; *p* < 0.001). Significant interactions between year and age group confirmed accelerated growth in elderly populations for both sexes.

**Figure 4:**
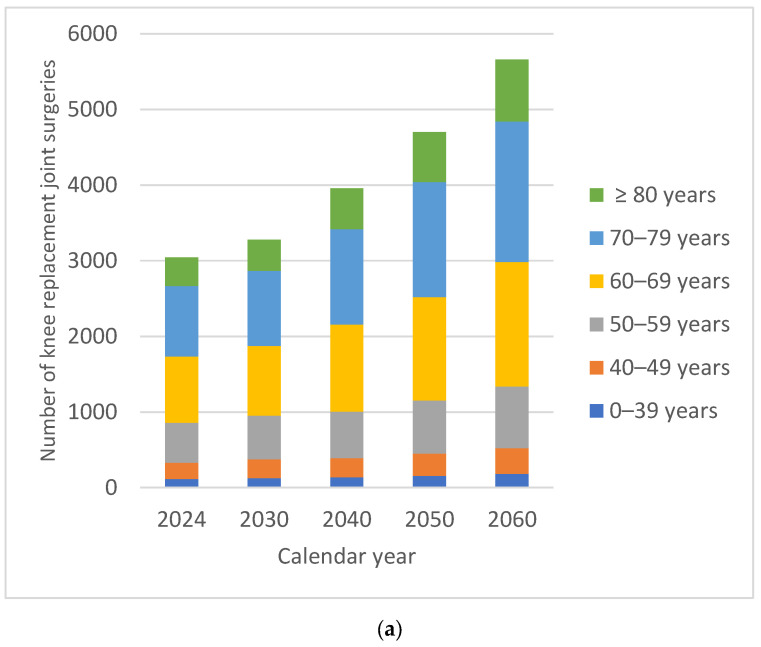

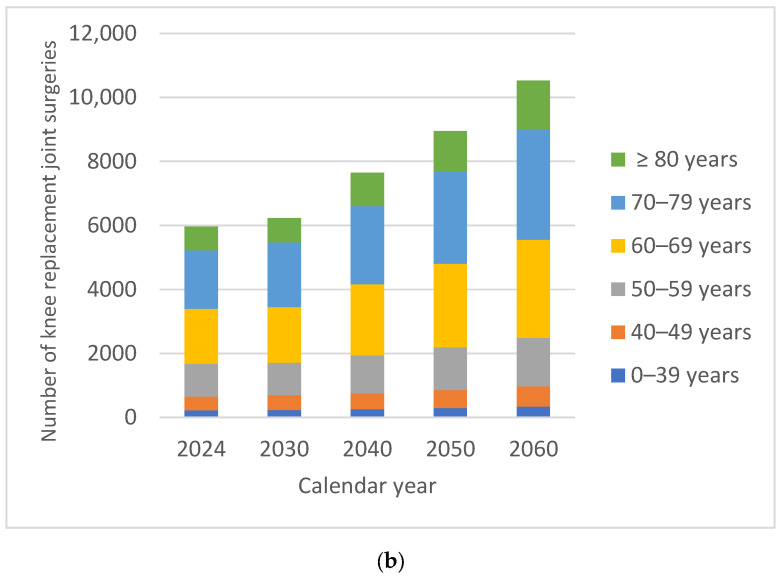
Projections for knee replacement joint surgeries to 2060: (**a**) In males; (**b**) In females. Uncertainty intervals for the ≥80 years age group are shown in Figure 2.

The projected overall numbers of KPjs were 9510 in 2030, 11,607 in 2040, 13,655 in 2050 and 16,186 in 2060. By 2060, there would be an estimated 79.78% increase in the number of KPjs compared with 2024. In males, the increase in the number of KPjs was 85.91% compared with 76.65% in females. In both males and females, the percentage of HPjs in 2060 recorded slightly smaller increases in patients under 60 years of age. For both males and females, the demand for surgical interventions was higher in patients over 60 years of age, on average 78% higher for males and 85% higher for females. The oldest patients, over 80 years of age, recorded the largest relative increase in volume over time, 115.83% for males and 105.26% for females, respectively (Figure 4).

In 2017 the RAR recorded 133 knee revision surgeries, and in 2024 it recorded 227 interventions. The projected overall numbers of KRjs were 244 in 2030, 341 in 2040, 401 in 2050 and 475 in 2060 (Table 6).

**Figure 5:**
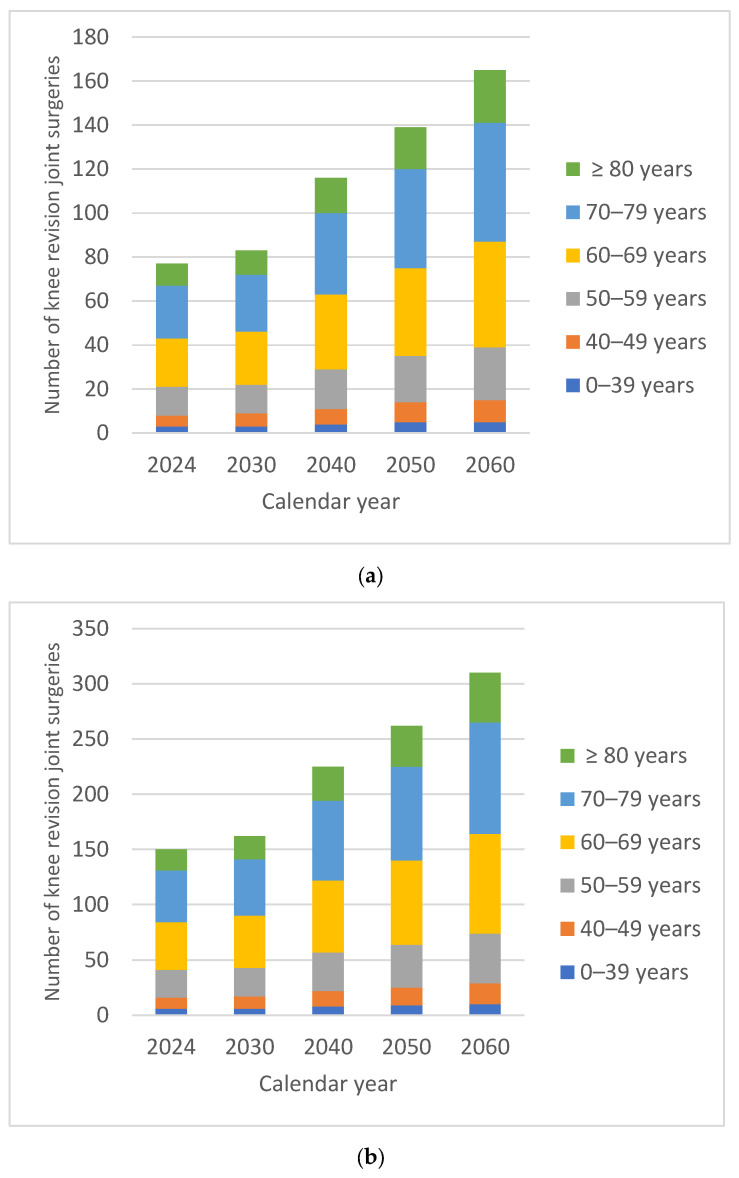
Projections for knee revision joint surgeries to 2060: (**a**) In males; (**b**) In females. Uncertainty intervals for the ≥80 years age group are shown in Figure 2.

By 2060, there would be an estimated 109.25% increase in the number of knee revision surgeries compared with 2024. In males, the increase in the number of KRjs was 115.58% compared with 106.00% in females. In both males and females, the percentage of KRjs in 2060 recorded slightly smaller increases in patients under 60 years of age (Figure 5).

For both males and females, the demand for surgical interventions was higher in patients over 60 years of age, on average 52% higher for males and 53% higher for females. The oldest patients, over 80 years of age, recorded the largest relative increase in volume over time, 140.00% for males and 136.84% for females, respectively.

The Incidence Rate Ratios (IRRs) are numerically computed with 95% confidence intervals, derived directly from the projections for hip and knee replacements and revisions and national population data, using Poisson regression with population offsets (Table 7).

Poisson regression analysis adjusting for population size demonstrated statistically significant annual increases in all arthroplasty procedure rates between 2017 and 2024. Hip replacement joint surgery increased by 2.4% per year (IRR 1.024, 95% CI 1.021–1.027; *p* < 0.001), while knee replacement joint surgery exhibited a markedly steeper rise of 11.1% per year (IRR 1.111, 95% CI 1.107–1.116; *p* < 0.001).

Revision procedures also increased significantly over time, with hip revision surgery rising by 1.6% annually (IRR 1.016, 95% CI 1.004–1.028; *p* = 0.009) and knee revision surgery by 9.2% annually (IRR 1.092, 95% CI 1.068–1.117; *p* < 0.001). These findings confirm sustained growth in arthroplasty demand independent of demographic change.

It is important to note that these projections represent expected procedure volumes under the assumption that future healthcare capacity and access evolve similarly to the recent past. They do not estimate the full clinical need, which may be higher due to existing waiting lists and unmet demand.

### 3.3. Sensitivity and Robustness Analyses

Projection results were robust across alternative modeling assumptions. Use of optimistic and pessimistic population scenarios altered projected 2060 procedure volumes by −11.8% to +9.6% compared with the constant population variant, without changing overall growth patterns.

Models restricted to post-pandemic recovery years produced slightly higher annual growth rates for knee replacement surgery but resulted in projected volumes within the 95% prediction intervals of the primary model.

Analysis of revision-to-primary ratios showed no statistically significant temporal trend for either hip or knee procedures (*p* = 0.42 and *p* = 0.37, respectively), supporting the assumption that revision volumes scale proportionally with primary procedures.

Under alternative growth scenarios, projected 2060 volumes varied within plausible ranges (Table 8).

The conservative deceleration scenario produced 11–17% lower projections than the primary linear model, while the accelerated growth scenario yielded 13–20% higher projections. The log-linear model, which assumes constant exponential growth, generated projections 5–11% higher than the linear model. Notably, all scenarios confirmed the core findings: substantial increases in all procedure types by 2060, with knee procedures (both replacement and revision) growing more rapidly than hip procedures, and the oldest age group (≥80 years) experiencing the largest relative increases. The consistency of these patterns across scenarios supports the robustness of our primary conclusions despite uncertainty in exact growth trajectories.

**Figure 6:**
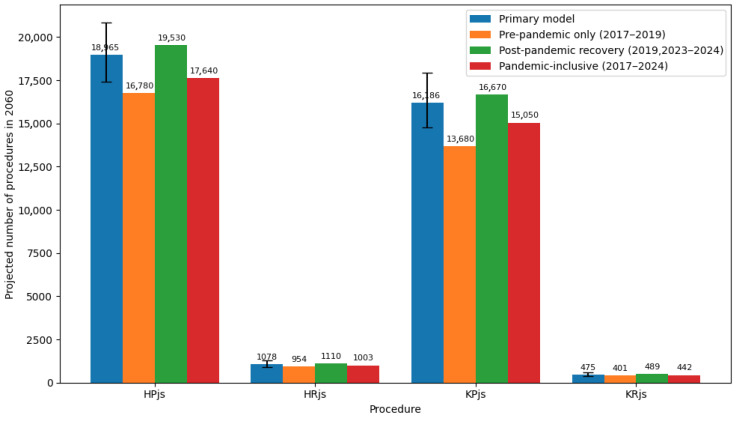
Comparison of projected 2060 procedure volumes under different pandemic-related data inclusion strategies. Error bars represent 95% prediction intervals for the primary model.

We also performed an analysis of the pandemic’s impact on projections (Figure 6).

Models restricted to pre-pandemic data (2017–2019) produced lower annual growth rates, particularly for knee procedures (KPjs: IRR 1.076 vs. 1.111 in primary model). When applied to 2060 projections, the pre-pandemic-only model yielded volumes 8–15% lower than the primary model for hip procedures and 12–19% lower for knee procedures. Conversely, models including pandemic years (2020–2022) produced artificially depressed growth rates due to service interruptions, underestimating 2060 demand by 5–9%. The post-pandemic recovery model (2019, 2023–2024) generated slightly higher growth rates than the primary model, particularly for knee replacement (IRR 1.124 vs. 1.111), but resulted in projected volumes within the 95% prediction intervals of the primary model. These findings confirm that excluding the pandemic disruption years while including the post-pandemic recovery years provides a balanced estimate that avoids both the underestimation from pandemic-inclusive models and the potential underestimation of recent demand acceleration captured in 2023–2024 data.

The analysis of revision-to-primary ratios revealed that over the study period (2017–2024, excluding pandemic years), the hip revision-to-primary ratio ranged from 0.055 to 0.060 (mean 0.058, 95% CI 0.056–0.060). The knee revision-to-primary ratio ranged from 0.025 to 0.031 (mean 0.028, 95% CI 0.026–0.030). Poisson regression with revisions as the outcome and primary procedures as the offset showed no statistically significant annual change for either hip (IRR 0.997, 95% CI 0.988–1.006; *p* = 0.52) or knee (IRR 1.009, 95% CI 0.993–1.025; *p* = 0.27) revision-to-primary ratios. These findings indicate that the proportion of revisions relative to primary procedures remained stable over the observation period, supporting the use of primary procedure trends as a driver for revision volume projections.

Regarding the comparison of revision projection models, the trend-independent model, using revision-specific growth rates, projected a hip revision volume of 1112 (95% CI 920–1340) and a knee revision volume of 492 (95% CI 390–620) (Table 9). These estimates were within 3–5% of the primary model projections (1078 and 475, respectively). The 10-year lagged primary model produced slightly lower estimates for hip revisions (1034) and slightly higher for knee revisions (498), reflecting differences in the temporal trajectory of primary volumes. The constant ratio with bootstrap uncertainty yielded 95% prediction intervals (hip: 890–1310; knee: 370–610) that overlapped substantially with the primary model intervals.

Across all alternative models, the relative increase from 2024 to 2060 remained substantial (hip: 38–45%; knee: 102–115%), and the disproportionate growth in the ≥80 age group persisted (age-specific ratios remained consistent because age distributions of revisions mirrored those of primaries). These findings confirm that the core conclusions are robust to different revision modeling assumptions.

The prediction intervals for the age groups of interest, particularly the ≥80 years category summarizing projections for the ≥80 age group across all procedure types, including point estimates and 95% prediction intervals from the bootstrap, as well as scenario ranges from optimistic/pessimistic population forecasts is presented in Table 10.

For patients aged ≥80 years, the projected increases remain substantial across all uncertainty bounds (Figure 2).

For example, hip replacement in females is projected to reach 1480 in 2060 (95% PI 1350–1630), representing a 50.1% increase from 2024 even under the lower bound. Under the pessimistic population scenario, the estimate for this group is 1395 (a 41.5% increase), while under the optimistic scenario it reaches 1570 (a 59.2% increase). Similar patterns are observed for all procedure types and both sexes, confirming that the finding of disproportionately high growth in the oldest age group is robust to uncertainty in both statistical estimation and demographic assumptions.

In terms of population-based rates, primary hip replacement would increase from 69.0 per 100,000 population in 2024 to 123.2 per 100,000 in 2060—a 78.6% increase in the rate. For primary knee replacement, the rate would rise from 45.9 per 100,000 in 2024 to 105.2 per 100,000 in 2060—a 129.2% increase. These rate increases exceed the volume increases because the population is projected to decline over the same period (from 19.6 million in 2024 to 15.4 million in 2060). Table 11 presents the projected volumes alongside population-based rates, illustrating that the aging and shrinking population amplifies the increase in procedure rates beyond the raw volume increases.

These rate increases exceed the volume increases because Romania’s total population is projected to decline by approximately 21.5% over the same period (from 19.6 million in 2024 to 15.4 million in 2060). Thus, the same or even slightly increasing procedure volumes would translate into higher population-based rates simply due to a shrinking denominator. The fact that volumes are projected to increase substantially while the population declines creates a ‘double effect’: the aging population increases the at-risk population for arthroplasty, while the shrinking overall population amplifies the rate increase when expressed per 100,000 population.

### 3.4. Comparison with United Kingdom Projections

**Figure 7:**
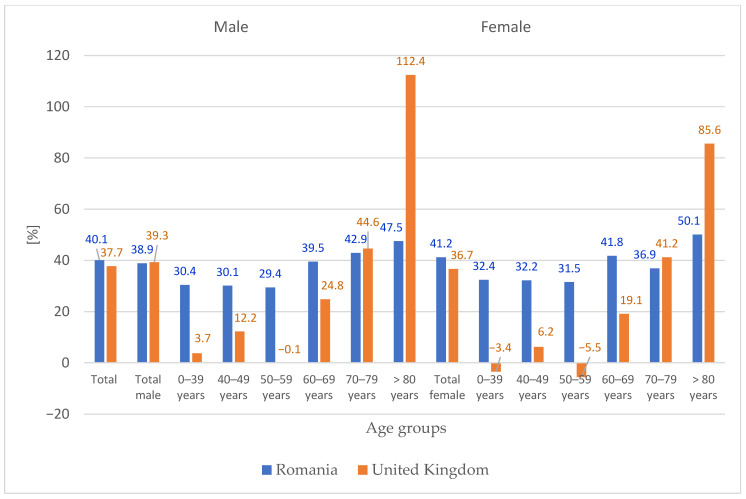
Comparison of the model results in Romania and United Kingdom, indicating the percentage change 2060 versus 2024 in hip replacement joint surgery by gender and age groups.

Figure 7 compares the projected percentage change (2060 vs. 2024) in primary hip replacement surgery between Romania and the United Kingdom (England, Wales, Northern Ireland, and the Isle of Man), stratified by age group and sex. While total projected increases are comparable between the two countries (Romania: 40.1%; UK: approximately 38%), notable differences emerge in the age distribution. In Romania, projections for age groups up to 69 years are higher than in the UK, whereas for the ≥80 age group, the UK shows substantially higher projected growth (UK: approximately 65% for males and 70% for females; Romania: 47.5% for males and 50.1% for females).

**Figure 8:**
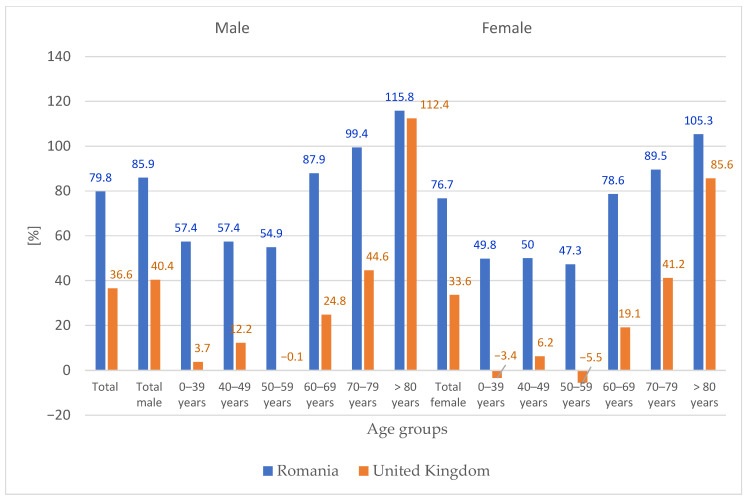
Comparison of the model results in Romania and United Kingdom, indicating the percentage change 2060 versus 2024 in knee replacement joint surgery by gender and age groups.

Figure 8 presents the corresponding comparison for primary knee replacement surgery. Romania’s projected total increase (79.8%) is approximately 2.2 times higher than the UK’s (approximately 36%). This difference is consistent across all age groups up to 79 years, with Romania showing markedly higher growth in every stratum. Only in the ≥80 age group do the projections converge (Romania: 115.8% for males, 105.3% for females; UK: approximately 110% for males, 105% for females).

These comparisons suggest that while both countries face increasing arthroplasty demand, Romania is currently on a steeper trajectory, particularly for knee replacement and among younger age groups. The convergence in the oldest age group may reflect a ‘catching up’ phenomenon as Romania’s healthcare system expands access to the very elderly, who have historically been underserved relative to their UK counterparts.

## 4. Discussion

This study projects substantial increases in all arthroplasty procedure types in Romania by 2060: primary hip replacement by 40.1% (from 13,526 in 2024 to 18,965), hip revision by 42.0% (from 759 to 1078), primary knee replacement by 79.8% (from 9003 to 16,186), and knee revision by 109.3% (from 227 to 475). The most striking finding is the disproportionate growth among patients aged ≥80 years, with projected increases ranging from 47.5% (male hip replacement) to 140.0% (male knee revision). These projections reflect both demographic aging and continued increases in age-specific procedure rates, with knee procedures growing more rapidly than hip procedures across all age strata. When expressed as population-based rates, the increases are even more pronounced: primary hip replacement rates per 100,000 population would rise by 78.6% and primary knee replacement rates by 129.2%, reflecting the combined effect of rising procedure volumes and a declining total population.

The important finding of our study is the increase in demand among patients of both sexes over 60 years of age and especially the very elderly, over 80 years of age. The risk of medical and surgical complications after replacement hip joint surgery, including mortality, increases significantly with patient age [48]. Thus, in the next 10 years of life, elderly patients have a 40% higher predisposition for complications [49,50]. When planning the future medical care of elderly patients, this increased risk should be considered.

The provision of elective surgical care has been massively disrupted worldwide by the COVID-19 pandemic. In Romania, the two waves of COVID-19 imposed national restrictions, which led to prolonged periods without elective surgeries, which were followed by periods with a reduced number of surgeries. Compared to 2019, volumes decreased in 2020 by 4103 hip surgeries (which represents 33.5%) and by 2479 knee surgeries (43.4%). It took approximately two years to reach pre-pandemic levels of intervention. In this short period of time, healthcare costs have increased substantially.

Different projection models have been used in the literature for hip and knee joint surgery. For example, Dixon et al. [51] used descriptive statistics and regression modelling of data obtained from hospitals. They assumed a continuous linear trend of historical data to predict future growth in the number of surgeries. Hooper et al. [15] used Poisson regression analysis combined with census data to make assumptions about past trends in surgical prevalence to determine whether it will continue to increase or remain constant. Culliford et al. [12] used a log-linear rate extrapolation over the prior period to determine future intervention projections. These data-based estimates have as their main limitation the absence from the model of hospital constraints related to the planning and surgeon intervention capacity, availability of operating rooms, hospital beds, etc. By formally quantifying temporal trends and incorporating uncertainty through prediction intervals and sensitivity analyses, the present study strengthens the evidence base for long-term arthroplasty planning in Romania. The robustness of findings across alternative demographic and modeling assumptions supports the validity of the projected increases, particularly among the very elderly population.

Our findings have concrete implications for multiple stakeholders in the Romanian healthcare system: Workforce planning—the projected 40–109% increases in surgical volumes imply a need for proportional expansion of the orthopaedic surgical workforce; Hospital capacity and infrastructure—operating theatre capacity will need to expand by approximately 50–100% depending on procedure mix. This translates to an estimated need for 20–30 new dedicated arthroplasty operating rooms by 2040, plus associated inpatient beds and intensive care capacity. Hospital administrators should incorporate these projections into capital planning cycles; Budgeting and reimbursement—the projected volume increases imply proportionate growth in procedure-related expenditures. Under current average reimbursement rates, total annual arthroplasty expenditure could rise from approximately €65 million in 2024 to €115–130 million by 2060 [52]; Implant supply chain—implant manufacturers require 5–10-year lead times to scale production capacity. Our projections provide a basis for forecasting demand for different implant types (e.g., cemented vs. uncemented, conventional vs. patient-specific), enabling supply chain planning to prevent shortages; Priority populations—the disproportionate growth in the ≥80 age group highlights the need for enhanced perioperative care pathways for frail elderly patients, including geriatrician involvement, comprehensive preoperative assessment, and postoperative rehabilitation services.

The disproportionate growth projected for patients aged ≥80 years (47.5–140.0% increases by 2060) carries specific implications for reimbursement policy and insurance design. Elderly patients undergoing arthroplasty have higher rates of medical and surgical complications, longer hospital stays, increased readmission risk, and greater postoperative care needs compared to younger populations. Under Romania’s current DRG-based reimbursement system, where hospitals receive a fixed payment per procedure regardless of patient complexity, this concentration of high-risk elderly patients may create financial disincentives for hospitals to treat the oldest old or may lead to ‘cream-skimming’ where healthier patients are prioritized. Such behaviors could exacerbate access disparities for the very elderly, precisely the population where need is growing fastest. To address this, policymakers could consider risk-adjusted DRG tariffs that provide higher reimbursement for patients aged ≥80 years or those with specific comorbidity profiles, reflecting their higher expected resource utilization.

The comparison with UK projections [47] reveals both similarities and divergences that merit discussion. The steeper growth trajectory for knee replacement in Romania across nearly all age groups (Figure 8) may reflect a later entry into the ‘arthroplasty epidemic’ phase, with Romania still experiencing rapid adoption of knee replacement as a treatment for osteoarthritis, whereas the UK may be approaching saturation in some age groups. The higher projections for younger age groups (40–69 years) in Romania compared to the UK could indicate expanding surgical indications to younger patients, a trend that has been observed in many countries as implant durability improves and patient expectations rise. Conversely, the lower projections for the ≥80 age group in Romania (particularly for hip replacement) may signal remaining access barriers for the very elderly, who may be under-referred for surgery due to concerns about perioperative risk or limited intensive care capacity. As Romania’s healthcare system continues to develop, this age group may experience accelerated growth beyond our current projections if access barriers are reduced. These international comparisons underscore the importance of context-specific projections and caution against directly applying findings from one country to another.

A crucial consideration when interpreting our projections is that they are based on performed procedures, not on the underlying clinical need. Romania, like many countries, experiences long waiting times for elective hip and knee arthroplasty [18], indicating that current procedure volumes are constrained by surgical capacity rather than reflecting full demand. During the waiting period, patients often experience deterioration in quality of life, increased pain, and functional decline [29,30,31], underscoring the gap between need and delivery.

The presence of capacity constraints has two important implications for our projections. First, the historical procedure rates used to estimate trends may be biased downward relative to true demand, because they reflect only those surgeries that the system could accommodate. If capacity constraints persist or worsen, future performed volumes may increase more slowly than demographic trends alone would suggest—potentially even falling short of our projections. Conversely, if the healthcare system expands capacity (e.g., through increased funding, more operating rooms, or greater surgical workforce), performed volumes could rise more rapidly, potentially catching up with unmet need and exceeding our projections. The net effect depends on policy decisions and investments in orthopaedic services.

Second, the observed trends (e.g., the rapid growth in knee replacement rates) could be influenced by changes in capacity over the study period. For instance, if hospitals gradually increased their arthroplasty volume in response to growing demand, the observed growth rate might partly reflect capacity expansion rather than purely demand-driven changes. Our model assumes that the average annual rate of change observed in 2017–2024 will continue, implicitly incorporating any ongoing capacity expansion into the projection. However, sudden policy shifts (e.g., major funding increases or new efficiency measures) could alter this trajectory.

To provide some insight into the potential magnitude of unmet need, we note that waiting lists for arthroplasty in Romania are reported to range from several months to over a year [18]. A rough estimate suggests that if waiting lists could be eliminated, the annual procedure volume might temporarily increase by 10–20% to clear the backlog, after which it would settle at a level closer to true demand. However, without precise waiting list data stratified by procedure and region, it is difficult to quantify this effect. Future research should aim to integrate waiting list dynamics into projection models to better distinguish between supply-constrained volumes and underlying need.

In summary, our projections should be viewed as a baseline scenario of what the healthcare system might deliver under continuation of recent trends in capacity and access. They likely represent a conservative estimate of true demand. Policymakers should consider both the projected volumes and the existing gap between need and delivery when planning future resources.

We acknowledge that projecting surgical demand 36 years into the future using only five observation points carries inherent uncertainty. While statistical tests supported linear trends over the study period, we cannot exclude the possibility of non-linear long-term trajectories driven by factors such as changing obesity prevalence, advances in implant longevity, shifts in surgical indications, or healthcare capacity constraints. Our sensitivity analyses address this uncertainty by demonstrating that while the exact magnitude of increase varies under different growth assumptions (conservative deceleration: 11–17% lower; accelerated growth: 13–20% higher), the core findings—substantial increases in all procedure types, disproportionately rapid growth in knee procedures, and the oldest age group experiencing the largest relative increases—remain robust across all scenarios. The prediction intervals and scenario ranges provided offer a quantitative sense of this uncertainty, but they cannot capture all possible future states. Therefore, these projections are best used as planning benchmarks that should be regularly updated as new registry data become available, rather than as fixed forecasts.

Our approach of projecting revision volumes based on primary procedure trends was supported by empirical analysis showing stable revision-to-primary ratios over the study period. Furthermore, sensitivity analyses using alternative revision models (independent trend, lagged primary, and constant ratio with bootstrap uncertainty) produced projections within the prediction intervals of the primary model, indicating that the choice of revision projection method does not materially alter the main conclusions. However, we acknowledge that a true cohort-based survivorship model would provide more accurate estimates if detailed implant-level data (e.g., time since primary, implant type, patient age at primary) were available. The Romanian Arthroplasty Register currently does not capture these variables in a format suitable for such analysis. As registry data mature, future studies should incorporate implant survival curves to refine revision projections.

The disproportionately high growth in patients aged ≥80 years, which is the central finding of this study, persists even when uncertainty is considered. Prediction intervals for this age group indicate that the lower bounds still represent substantial increases (e.g., 41.5% for female hip replacement), and scenario analyses using alternative population forecasts produce ranges that do not alter the directional conclusion. This robustness strengthens the policy implication that healthcare planners must prepare for a significant increase in demand among the very elderly.

An important consideration when interpreting long-term projections is that they are based on historical patterns of surgical practice, including the indications for surgery that prevailed during the observation period (2017–2024). However, surgical indications for hip and knee arthroplasty are not static; they evolve over time due to factors such as changing patient expectations, advances in implant technology, shifts in clinical guidelines, and the growing evidence base supporting arthroplasty in younger or older patients. For example, if the threshold for offering surgery continues to lower—offering TKA to patients with milder symptoms or to younger individuals with higher functional demands—the demand for primary procedures could accelerate beyond the rates observed in our study period. Conversely, if non-operative management (e.g., weight loss programs, novel disease-modifying drugs) becomes more effective, demand growth might decelerate.

Our projections assume that the average annual rates of change observed in 2017–2024 will continue, implicitly incorporating the net effect of these factors as they manifested during that period. However, if the pace of change in indications or implant survivorship accelerates or decelerates, the actual trajectory may deviate from our estimates [53]. For instance, if the trend toward operating on younger patients observed in many developed countries intensifies in Romania, the age distribution of primary procedures could shift downward, potentially altering the future revision burden. While our sensitivity analyses using alternative growth scenarios (conservative and accelerated) provide some insight into the range of possible outcomes, they do not explicitly model changes in indication mix. Future research should aim to incorporate data on patient age, comorbidity, and disease severity at the time of primary surgery to better predict how evolving indications might shape long-term demand.

Several real-world factors could modify the trajectory of arthroplasty demand and should be considered when interpreting our projections. First, healthcare policy changes—such as increased funding for elective surgery, expansion of day-case arthroplasty, or implementation of national procurement programs for implants—could accelerate growth by reducing waiting lists and expanding surgical capacity. Conversely, budget constraints, workforce shortages, or facility closures could dampen growth. Second, epidemiological shifts in obesity prevalence, which is a strong risk factor for osteoarthritis, could alter the underlying disease burden. If current trends toward rising obesity continue, demand could exceed our projections; if public health interventions successfully reduce obesity, demand could moderate. Third, advances in implant technology and surgical techniques could extend implant longevity, potentially reducing the long-term revision burden even as primary procedures increase. Fourth, the emergence of disease-modifying osteoarthritis drugs or effective non-surgical interventions could delay or obviate the need for arthroplasty, particularly in younger patients. Fifth, changing patient expectations and thresholds for surgery—influenced by direct-to-consumer advertising, social norms, and cultural attitudes toward aging and disability—could shift demand independently of disease prevalence. Finally, macroeconomic conditions and their impact on healthcare budgets and patients’ ability to access private care could introduce volatility. Our sensitivity analyses explored the impact of varying growth rates (conservative deceleration, accelerated growth) and alternative revision models, providing a range of plausible outcomes that bracket many of these uncertainties. However, the projections should be viewed as baseline scenarios under continuation of recent trends, not as definitive forecasts.

This study has several limitations. First, projections are based on only five years of complete registry data (excluding pandemic years), which limits the precision of long-term trend estimation. While statistical testing supported linear trends over the observation period, and sensitivity analyses across alternative growth scenarios confirmed the robustness of core findings, the exact numerical projections should be interpreted with appropriate caution given the 36-year forecast horizon.

Second, the Romanian Arthroplasty Register, while comprehensive in coverage, has limited data granularity compared to some international registries. Age-stratified analyses were constrained by available data categories, and information on comorbidities, obesity, and other patient-level factors that influence surgical demand was not available for inclusion in the model.

The Romanian Arthroplasty Register, while achieving high completeness for procedure counts (>97% for primary procedures), has limitations in the depth of clinical data collected. Specifically, the registry does not routinely capture: (1) patient comorbidities, which are important predictors of surgical risk and outcomes; (2) body mass index (BMI), a key risk factor for osteoarthritis and postoperative complications; (3) detailed indications for revision surgery (e.g., aseptic loosening, infection, instability, periprosthetic fracture), which would enable modeling of revision burden by cause; (4) implant-specific characteristics (bearing surface, fixation method, manufacturer), which could inform projections of implant survival; (5) procedures performed in the private sector are captured through mandatory reporting, but completeness may be slightly lower than in the public sector due to reporting delays; and (6) socioeconomic variables, which could help identify disparities in access to care. The absence of these variables precludes more nuanced modeling—for example, projecting revision demand using implant-specific survival curves or estimating the impact of obesity trends on future primary demand. Future enhancements to the registry should prioritize collecting these variables to enable more sophisticated projection models and to support health equity research.

Third, our projections do not account for potential future changes in surgical indications, implant longevity, or the emergence of disease-modifying treatments that could alter the need for arthroplasty. Similarly, healthcare system capacity constraints (surgeon workforce, hospital beds, operating room availability, funding limitations) are not incorporated into the model, meaning our projections represent estimated demand rather than realized supply.

Fourth, the COVID-19 pandemic created unprecedented disruptions that may have altered long-term trends in ways not fully captured by our modeling approach. While we explicitly tested the sensitivity of projections to different pandemic-related data inclusion strategies and found consistent directional patterns, residual uncertainty remains.

Fifth, the model does not account for potential future public health interventions, changes in obesity prevalence, or shifts in population health that could accelerate or decelerate arthroplasty demand beyond demographic trends and observed growth rates.

Sixth, our projections are based on registry counts of performed procedures, which are subject to healthcare system capacity constraints and waiting lists. As such, they do not represent true clinical demand, which is likely higher. The model assumes that the relationship between population characteristics and performed procedures remains stable, but this relationship could change if capacity expands or contracts. The presence of waiting lists introduces uncertainty about the extent to which past trends reflect changes in demand versus changes in supply. Without detailed data on waiting lists and unmet need, we cannot quantify this bias, but we note that it likely leads to an underestimation of future demand if capacity remains unchanged, and an overestimation relative to what could be achieved if capacity were to shrink.

While we have provided uncertainty intervals for age/sex subgroups using bootstrap methods, these intervals do not capture all potential sources of uncertainty, such as future changes in age-specific procedure rates (e.g., if the propensity for surgery among the very elderly changes over time). However, sensitivity analyses using alternative population scenarios provide a complementary assessment.

Several avenues for future research emerge from this study: Registry enhancement—Expanding the Romanian Arthroplasty Register to include patient-reported outcomes, comorbidities, BMI, and detailed revision indications would enable more sophisticated projection models incorporating implant survival curves and risk-adjusted demand forecasts; Microsimulation modelling—Developing individual-level microsimulation models that track patients from disease onset through primary surgery to revision and death would allow incorporation of competing risks and time-varying covariates; Capacity-constrained projections—Integrating our demand projections with discrete event simulation models of hospital capacity (operating rooms, beds, staff) could generate more realistic projections of achievable volumes under different investment scenarios; Sub-national projections—Regional variation in population age structure, disease prevalence, and healthcare access could be modeled to inform decentralized planning; International comparative research—Replicating our methodology in other Central and Eastern European countries would reveal whether Romania’s trajectory is unique or reflects regional patterns, facilitating cross-border learning and potential collaborative planning; Health equity analyses—Linking registry data with socioeconomic indicators would enable projection of disparities in arthroplasty access and outcomes, informing targeted interventions; Economic evaluations—Incorporating our volume projections into cost-effectiveness models of arthroplasty versus non-surgical management would help optimize resource allocation as demand grows [54,55].

## 5. Conclusions

This study projects substantial growth in arthroplasty procedures in Romania over the next four decades, with primary knee replacement nearly doubling and knee revision more than doubling by 2060 relative to 2024 levels. The most clinically significant finding is the disproportionate increase among patients aged ≥80 years, who will account for an increasing share of surgical volume and for whom perioperative risk, resource utilization, and postoperative care needs are highest. These projections translate into concrete challenges for the Romanian healthcare system: the need to expand surgical workforce training (an estimated 10–15 additional arthroplasty surgeons annually), increase operating theatre capacity (20–30 new dedicated rooms by 2040), secure sustainable implant supply chains, and develop enhanced perioperative pathways for frail elderly patients. These projections are based on the best available data—complete national registry records and official population forecasts—and have been subjected to extensive sensitivity analyses (alternative growth scenarios, revision models, population variants, and pandemic-related data strategies) that confirm the robustness of the directional findings. However, they should be interpreted as baseline scenarios under continuation of recent trends, not as definitive predictions. Key uncertainties include future healthcare policy decisions, capacity constraints (currently reflected in waiting lists), epidemiological shifts (e.g., obesity trends), advances in implant technology, and potential disease-modifying therapies that could alter surgical demand. They depend on key modelling assumptions—including linear continuation of observed growth rates, stability of revision-to-primary ratios, and accuracy of official population forecasts—that were empirically supported over the study period but may not hold over a 36-year horizon. For healthcare planners, these findings provide quantitative benchmarks for workforce development, infrastructure investment, and budget forecasting. For researchers, they highlight the need for enhanced registry data (including comorbidities, BMI, and revision indications) to enable more sophisticated microsimulation models that can incorporate implant survival curves, competing risks, and capacity constraints. Regular updating of these projections as new registry data become available will be essential to track deviations from expected trajectories and adjust plans accordingly. International comparisons, particularly with other Central and Eastern European countries, would help contextualize Romania’s trajectory and facilitate cross-border learning. Ultimately, these projections are a call to action: the aging of Romania’s population and the continued expansion of arthroplasty indications will inevitably increase demand, and proactive planning is essential to ensure that the healthcare system can meet this challenge equitably and safely.

## Figures and Tables

**Table 1:** Total number of patients in the period 2017–2024 who participated in the study.

Year	Hip Replacement Joint Surgery	Hip Replacement Joint Surgery Male	Hip Replacement Joint Surgery Female	Hip Revision Joint Surgery	Knee Replacement Joint Surgery	Knee Replacement Joint Surgery Male	Knee Replacement Joint Surgery Female	Knee Revision Joint Surgery
2017	11,705	4918	6787	719	4223	954	3269	133
2018	11,944	5063	6881	651	4894	1141	3753	147
2019	12,180	5282	6898	699	5718	1324	4394	171
2023	13,602	6078	7524	751	8445	2056	6389	265
2024	13,526	5845	7681	759	9003	2279	6724	227

**Table 2:** Population statistics in the study period.

Year	Population	Male Population	Female Population
2017	19,588,715	9,520,190	10,068,525
2018	19,473,970	9,464,350	10,009,620
2019	19,371,648	9,414,631	9,957,017
2023	19,118,479	9,291,581	9,826,898
2024	19,015,088	9,241,332	9,773,756
2030	18,359,507	8,919,721	9,439,786
2040	17,794,417	8,645,067	9,149,350
2050	16,027,266	7,788,247	8,239,019
2060	15,390,600	7,479,832	7,910,768

**Table 3:** Projections for hip replacement joint surgery up until 2060.

	Recorded Number of HPjs					Projected Number of HPjs				Percentage Change 2060 vs. 2024
Year	2017	2018	2019	2023	2024	2030	2040	2050	2060 (95%PI) ^1^	
Total	11,705	11,944	12,180	13,602	13,526	13,693 (12,950–14,450)	16,157 (15,200–17,200)	17,151 (16,100–18,300)	18,965 (17,420–20,830)	40.14
Total males	4916	5063	5283	6078	5848	5868	6956	7421	8123	38.93
0–39 years	186	191	200	230	214	215	239	255	279	30.37
40–49 years	349	359	375	431	402	416	448	478	523	30.10
50–59 years	849	875	913	1050	977	987	1082	1155	1264	29.37
60–69 years	1416	1459	1522	1751	1688	1686	2017	2152	2355	39.51
70–79 years	1504	1549	1617	1860	1815	1811	2221	2370	2593	42.87
≥80 years	612	630	657	756	751	753	949	1013	1108	47.53
Total females	6789	6881	6897	7524	7678	7825	9202	9730	10,843	41.22
0–39 years	257	260	261	284	281	286	316	334	372	32.38
40–49 years	481	488	489	533	528	536	593	627	698	32.19
50–59 years	1173	1189	1192	1300	1283	1297	1432	1514	1687	31.49
60–69 years	1956	1982	1987	2168	2217	2266	2668	2821	3144	41.81
70–79 years	2077	2106	2110	2302	2383	2405	2938	3107	3462	36.88
≥80 years	805	856	858	936	986	1035	1256	1328	1480	50.10

^1^ Prediction intervals are presented for total projected procedure volumes only. Age- and sex-specific projections are deterministic allocations based on population structure and therefore do not have independent uncertainty estimates.

**Table 4:** Projections for hip revision joint surgery up until 2060.

	Recorded Number of HRjs					Projected Number of HRjs				Percentage Change 2060 vs. 2024
Year	2017	2018	2019	2023	2024	2030	2040	2050	2060 (95%PI) ^1^	
Total	719	651	699	751	759	778 (756–800)	918 (848–988)	975 (854–1096)	1078 (910–1280)	42.02
Total males	312	282	303	325	328	332	395	422	462	40.85
0–39 years	12	11	11	12	12	13	14	14	15	25.00
40–49 years	22	20	21	22	23	22	25	26	28	21.73
50–59 years	54	49	52	54	55	56	61	63	67	21.82
60–69 years	90	81	87	94	95	95	115	123	134	41.05
70–79 years	95	86	93	101	102	103	126	137	151	48.04
≥80 years	39	35	38	42	42	43	54	59	67	59.52
Total females	407	369	396	426	431	446	523	533	616	42.92
0–39 years	15	14	15	16	16	17	18	18	20	25.00
40–49 years	29	26	28	29	30	31	34	34	37	23.33
50–59 years	70	64	68	71	72	73	81	83	89	23.61
60–69 years	117	106	114	123	124	130	152	161	179	44.35
70–79 years	125	113	121	132	134	138	167	179	202	50.75
≥80 years	51	46	49	55	55	57	71	78	89	61.81

^1^ Prediction intervals are presented for total projected procedure volumes only. Age- and sex-specific projections are deterministic allocations based on population structure and therefore do not have independent uncertainty estimates.

**Table 5:** Projections for knee replacement joint surgery up until 2060.

	Recorded Number of KPjs					Projected Number of KPjs				Percentage Change 2060 vs. 2024
Year	2017	2018	2019	2023	2024	2030	2040	2050	2060 (95%PI) ^1^	
Total	4223	4894	5718	8445	9003	9510 (9354–9666)	11,607 (11,100–12,114)	13,655 (12,686–14,624)	16,186 (14,780–17,960)	79.78
Total males	978	1133	1324	2855	3044	3281	3958	4701	5659	85.91
0–39 years	37	43	50	108	115	125	136	156	181	57.39
40–49 years	69	80	94	202	216	249	255	293	340	57.40
50–59 years	169	196	229	493	526	577	616	704	815	54.94
60–69 years	282	326	381	823	877	923	1148	1366	1648	87.91
70–79 years	299	347	405	874	931	995	1264	1522	1857	99.46
≥80 years	122	141	165	355	379	412	540	660	818	115.83
Total females	3245	3761	4394	5590	5959	6229	7649	8954	10,527	76.65
0–39 years	123	142	166	211	225	232	262	297	337	49.77
40–49 years	230	267	312	396	422	463	493	557	633	50.00
50–59 years	561	650	759	966	1030	1012	1190	1342	1517	47.28
60–69 years	935	1084	1266	1610	1717	1746	2218	2602	3066	78.56
70–79 years	993	1151	1345	1710	1823	2014	2442	2898	3454	89.46
≥80 years	404	468	547	595	741	762	1044	1258	1521	105.26

^1^ Prediction intervals are presented for total projected procedure volumes only. Age- and sex-specific projections are deterministic allocations based on population structure and therefore do not have independent uncertainty estimates.

**Table 6:** Projections for knee revision joint surgery up until 2060.

	Recorded Number of KRjs					Projected Number of KRjs				Percentage Change 2060 vs. 2024
Year	2017	2018	2019	2023	2024	2030	2040	2050	2060 (95%PI) ^1^	
Total	133	147	171	265	227	244 (235–253)	341 (307–375)	401 (337–465)	475 (380–590)	109.25
Total males	31	34	40	90	77	83	116	138	166	115.58
0–39 years	1	1	1	3	3	3	4	5	5	66.66
40–49 years	2	2	3	6	5	6	7	9	10	100.00
50–59 years	5	6	7	15	13	13	18	21	24	84.62
60–69 years	9	10	11	26	22	24	34	40	48	118.18
70–79 years	9	10	12	28	24	26	37	45	54	125.00
≥80 years	4	4	5	12	10	11	16	19	24	140.00
Total females	102	113	131	175	150	161	225	263	309	106.00
0–39 years	4	4	5	6	6	6	8	9	10	66.66
40–49 years	7	8	9	12	10	11	14	16	19	90.00
50–59 years	18	20	23	29	25	26	35	39	45	80.00
60–69 years	29	33	38	51	43	47	65	76	90	109.30
70–79 years	31	35	40	54	47	51	72	85	101	114.89
≥80 years	13	14	16	23	19	21	31	37	45	136.84

^1^ Prediction intervals are presented for total projected procedure volumes only. Age- and sex-specific projections are deterministic allocations based on population structure and therefore do not have independent uncertainty estimates.

**Table 7:** Incidence Rate Ratios for annual trends in hip and knee replacement and revision joint surgery in Romania (2017–2024).

Procedure	IRR per Year	95% Confidence Interval	*p*-Value
Hip replacement (HPjs)	1.024	1.021–1.027	<0.001
Hip revision (HRjs)	1.016	1.004–1.028	0.009
Knee replacement (KPjs)	1.111	1.107–1.116	<0.001
Knee revision (KRjs)	1.092	1.068–1.117	<0.001

**Table 8:** Comparison of projected 2060 procedure volumes under alternative growth scenarios.

Procedure	Primary Linear Model	Scenario A (Conservative)	Scenario B (Accelerated)	Scenario C (Log-Linear)
Hip replacement (HPjs)	18,965	16,843 (−11.2%) ^1^	21,427 (+13.0%)	19,842 (+4.6%)
Hip revision (HRjs)	1078	956 (−11.3%)	1218 (+13.0%)	1128 (+4.6%)
Knee replacement (KPjs)	16,186	13,432 (−17.0%)	19,437 (+20.1%)	17,954 (+10.9%)
Knee revision (KRjs)	475	394 (−17.1%)	571 (+20.2%)	527 (+10.9%)

^1^ Values in parentheses indicate percentage difference from primary model projections.

**Table 9:** Comparison of projected 2060 revision volumes under alternative modeling assumptions.

Procedure	Primary Model (Trend from Primary)	Independent Trend Model	Lagged Primary Model (10-Year)	Constant Ratio + Bootstrap (95% PI)
Hip revision (HRjs)	1078 (910–1280) ^1^	1112 (920–1340)	1034	1078 (890–1310)
Knee revision (KRjs)	475 (380–590)	492 (390–620)	498	475 (370–610)

^1^ Values in parentheses are 95% prediction intervals where available. Primary model intervals are from Table 4 and Table 6.

**Table 10:** Projected 2060 procedure volumes for patients aged ≥80 years: point estimates, 95% prediction intervals, and population scenario ranges.

Procedure	Sex	Point Estimate	95% Prediction Interval (Bootstrap)	Optimistic Population Scenario	Pessimistic Population Scenario
Hip replacement (HPjs)	Male	1108	1010–1220 ^1^	1175	1045
	Female	1480	1350–1630	1570	1395
Hip revision (HRjs)	Male	67	58–78	71	63
	Female	89	77–103	94	84
Knee replacement (KPjs)	Male	818	740–910	868	772
	Female	1521	1380–1680	1614	1435
Knee revision (KRjs)	Male	24	19–30	25	23
	Female	5	37–55	48	42

^1^ Bootstrap prediction intervals incorporate uncertainty in baseline rates and trends. Population scenario ranges reflect the optimistic and pessimistic variants of the national population forecast, assuming constant procedure rates and trends.

**Table 11:** Baseline (2024) and projected (2060) procedure volumes and rates per 100,000 population.

Procedure	2024 Volume	2024 Rate (per 100,000)	2060 Volume	2060 Rate (per 100,000)	Absolute Volume Increase	Relative Volume Increase	Relative Rate Increase
Hip replacement (HPjs)	13,526	69.0 ^1^	18,965	123.2 ^1^	5439	40.1%	78.6%
Hip revision (HRjs)	759	3.9	1078	7.0	319	42.0%	79.5%
Knee replacement (KPjs)	9003	45.9	16,186	105.2	7183	79.8%	129.2%
Knee revision (KRjs)	227	1.2	475	3.1	248	109.3%	158.3%

^1^ Population estimates: 19,618,996 in 2024; 15,390,600 in 2060 (Table 2). Rates calculated as (volume/population) × 100,000.

## Data Availability

Data is contained within the article.

## Abbreviations

The following abbreviations are used in this manuscript:

nHPjs	the volume of hip replacement joint surgery
nHRjs	the volume of hip revision joint surgery
nKPjs	the volume of knee replacement joint surgery
nKRjs	the volume of knee revision joint surgery
NP	number of populations
rHPjs	the rate for hip replacement joint surgery
rHRjs	the rate for hip revision joint surgery
rKPjs	the rate for knee replacement joint surgery
rKRjs	the rate for knee revision joint surgery
y	year
VT	variation trend
FPnHPjs	future projection for the volume of replacement hip joint surgeries
FPnHRjs	future projection for the volume of hip revision joint surgery
FPnKPjs	future projection for the volume of knee replacement joint surgery
FPnKRjs	future projection for the volume of knee revision joint surgery
NIS	National Institute of Statistics
RAR	Romanian Arthroplasty Register
